# Comparison of intravenous immunoglobulin and plasma exchange in hospital-acquired infections of autoimmune encephalitis in a tertiary care center

**DOI:** 10.2478/abm-2025-0018

**Published:** 2025-06-30

**Authors:** Totsapol Surawattanawong, Akarin Hiransuthikul, Panthicha Katasrila, Thiravat Hemachudha, Abhinbhen W. Saraya

**Affiliations:** Department of Medicine, Faculty of Medicine, Chulalongkorn University, Bangkok 10330, Thailand; Department of Preventive and Social Medicine, Faculty of Medicine, Chulalongkorn University, Bangkok 10330, Thailand; Thai Red Cross EID and Health Science Centre, King Chulalongkorn Memorial Hospital, The Thai Red Cross Society, Bangkok 10330, Thailand

**Keywords:** autoimmune encephalitis, hospital-acquired infections, intravenous immunoglobulin, plasma exchange

## Abstract

**Background:**

The prevailing approach for the acute-phase treatment of autoimmune encephalitis (AIE) is currently the administration of intravenous immunoglobulin (IVIG) or plasma exchange (PLEX), in conjunction with high-dose corticosteroids. Despite this, there is still no definitive evidence on the risks and benefits of IVIG vs. PLEX in terms of treatment-related complications.

**Objectives:**

The primary objective of this study was to determine the differences in the cumulative incidence of hospital-acquired infections (HAIs) in patients diagnosed with AIE, who received either IVIG or PLEX. The secondary objectives were to explore the differences in the duration of hospitalization and levels of disability.

**Methods:**

Patients who were hospitalized at the King Chulalongkorn Memorial Hospital, Thailand, due to AIE, were aged ≥15 years, and had received either IVIG or PLEX during their hospitalization from January 2015 to December 2020 were included in the study. The modified Rankin scale (mRS) was utilized to evaluate the degree of disability at admission and discharge.

**Results:**

Among the 44 patients included in the study, 10 (22.7%) received PLEX and 34 (77.3%) received IVIG. Those who received IVIG were significantly less likely to have HAIs (14.7% vs. 50.0%, *P* = 0.03) and had a significantly shorter duration of hospitalization (median [IQR] 12.0 [6.0 – 23.0] vs. 25.0 [21.0 – 49.0] d, *P* = 0.01) compared to those who received PLEX. Primary septicemia was the most commonly observed cause of infection in both groups. There were no significant differences in mRS at discharge, changes in mRS between admission and discharge, and the total direct cost of hospitalization between the two groups.

**Conclusions:**

The utilization of IVIG is associated with a diminished occurrence of nosocomial infections, leading to shorter hospitalization and potential cost benefits. Our findings propose that IVIG may represent a more beneficial therapeutic alternative for AIE patients compared with PLEX.

Autoimmune encephalitis (AIE) is a neurological disorder resulting from immune system activation and is currently recognized as the most common noninfectious cause of encephalitis. The pathophysiology of AIE is characterized by the presence of two types of antibodies: those targeting intracellular antigens and those targeting neuronal surface antigens [1,2,3]. Intracellular antibodies stimulate the activity of cytotoxic T-cells and can serve as a surrogate marker for disease activity. However, these antibodies are generally less responsive to antibody depletion therapy when compared with neuronal surface antibodies [4, 5].

The current standard treatment for AIE typically involves combination therapy, consisting of the administration of a corticosteroid alongside either plasma exchange (PLEX) or intravenous immunoglobulin (IVIG). Corticosteroids are known to have a greater impact on reducing the activity of T cells rather than B cells, which are considered to be the primary mechanism underlying the pathogenesis of AIE, especially in the neuronal surface antibody-mediated group. As a result, many patients with AIE only experience partial responses to corticosteroid treatment and require antibody depletion therapy as an additional treatment [5,6,7].

IVIG is a form of immunoglobulin G (IgG) that can inhibit the activity of antibodies present in the bloodstream via the process of autoantibody neutralization [8]. Nonetheless, the process of antibody removal may lead to certain infections. In 1994, the Food and Drug Administration (FDA) and the Centers for Disease Control and Prevention (CDC) reported an incidence of hepatitis C infection associated with the use of IVIG. However, no other specific incidents of infection related to IVIG therapy have been reported since that time [9]. In addition, IVIG therapy has been associated with various side effects, including flu-like symptoms, headache, hypotension, and severe allergic reactions (particularly in patients with IgA deficiency) [10].

Since 1978, PLEX has been primarily used to treat Guillain-Barré syndrome (GBS), as it removes antibodies from the bloodstream and may cause a slight reduction in white blood cell count [11]. Subsequently, PLEX has also been used for other immune-mediated neurological diseases, including AIE. The adverse effects associated with PLEX are anaphylaxis, hypokalemia, and transfusion-related acute lung injury [12, 13]. Moreover, the depletion of immunoglobulins can increase the risk of opportunistic infections [14] and lead to the removal of certain medications [15].

In 1991, Pohl et al. [16] analyzed the prevalence of infection among individuals with severe diffuse proliferative lupus nephritis, who underwent treatment with high-dose corticosteroids and immunosuppressive medication, in comparison to those who received PLEX and immunosuppressive medication. The incidence of infection in both treatment groups was found to be non-significantly divergent. Several years later, Mokrzycki and Kaplan [17] undertook a study to evaluate the safety of PLEX, assessing 699 treatments in patients diagnosed with Goodpasture’s syndrome, multiple myeloma, cryoglobulinemia, and thrombotic thrombocytopenic purpura. Their findings revealed an infection rate of 0.02%. Thereafter, no definitive studies regarding PLEX-associated infections were reported until 2019, when Lu et al. [18] performed a retrospective analysis that revealed an infection rate of 0.35%, with two-thirds being catheter-related infections.

A 1995 study investigated the duration of hospitalization for mild GBS in patients treated with PLEX and IVIG. The results of this study demonstrated that the IVIG treatment group experienced a significantly shorter duration of hospital stay (IVIG = 8.5 ± 4.4 d, PLEX = 13.6 ± 6.7; *P* = 0.05) [19]. It is noteworthy, however, that the disparity may be attributed to the longer treatment course of the PLEX arm. Furthermore, the patients recruited in this study had mild GBS and were therefore less severely affected than the average AIE patient.

To our knowledge, a comparative assessment of the incidence of nosocomial infections and hospital stay (irrespective of treatment duration) between AIE patients undergoing PLEX and those receiving IVIG has yet to be conducted. Given that several public healthcare insurance schemes worldwide only provide coverage for PLEX, not IVIG, we propose undertaking a study to appraise these critical issues and determine the superior therapeutic modality. The primary objective of this study was to determine the differences in the cumulative incidence of hospital-acquired infections (HAIs) in patients diagnosed with AIE who received either IVIG or PLEX. The secondary objectives were to explore the differences in the duration of hospitalization and in the levels of disability.

## Methods

This study was approved by the Institutional Review Board of the Faculty of Medicine, Chulalongkorn University (IRB No. 046/63). All potential participants provided written informed consent, thereby confirming their voluntary participation in the research.

### Enrollment of participants

A combined retrospective and prospective cohort study of adult patients hospitalized due to AIE at King Chulalongkorn Memorial Hospital (KCMH), The Thai Red Cross Society, has been conducted since January 2015. KCMH is a tertiary care center and serves as the teaching hospital for the Faculty of Medicine, Chulalongkorn University, located in Bangkok, Thailand. All AIE patients seen by the Neuroinflammation Unit between January 2015 and December 2020 were included. The inclusion criteria were patients aged ≥15 years who fulfilled the diagnostic criteria for AIE, proposed by Graus et al. [20], and received IVIG or PLEX. The exclusion criteria included individuals who did not receive concomitant 1 g/d of intravenous pulse methylprednisolone (IVMP) for 3–5 d during admission, had a history of prolonged exposure to neurotoxic substances or illicit drugs, or were pregnant. The choice of treatment was at the discretion of the attending physician and based on the patient’s family preference. We used retrospective data collected between January 2015 and December 2019 and prospectively enrolled patients from January 2020 to December 2020.

#### Statistical analysis

The outcomes of the study included the occurrence of HAIs, duration of hospitalization, and degree of disability at discharge. HAIs were categorized into 5 categories as follows: severe (septic shock), moderate-to-severe (severe sepsis), moderate (sepsis), mild-to-moderate (no sepsis but required intravenous antibiotics), and mild (no sepsis but required oral antibiotics). The definitions of septic shock, severe sepsis, and sepsis were based on the Surviving Sepsis Campaign’s International Guidelines for Management of Severe Sepsis and Septic Shock 2012 [21]. The modified Rankin scale (mRS) was utilized to evaluate the degree of disability at admission and discharge.

Demographic and hospitalization information were summarized as median (interquartile range [IQR]) and number (percentage) for continuous and categorical variables, respectively. A histogram and Shapiro–Wilk test were used to assess the normality of the data. Characteristics between patients receiving IVIG and PLEX were compared using Pearson’s chi-square, Fisher’s exact test, or the Mann–Whitney test, as appropriate. A multivariable logistic regression model was used to evaluate the association between treatment groups and the occurrence of HAIs, adjusting for age, sex, and concurrent use of immunosuppressive drugs. Statistical significance was defined as *P* < 0.05. All statistical analyses were performed using Stata/SE 17.0 (Stata-Corp LP).

## Results

Among 57 AIE patients according to the criteria of Graus et al. [20] from the KCMH adult database, 52 patients were retrospectively collected, and 5 patients were prospectively selected.

**Figure 1.**
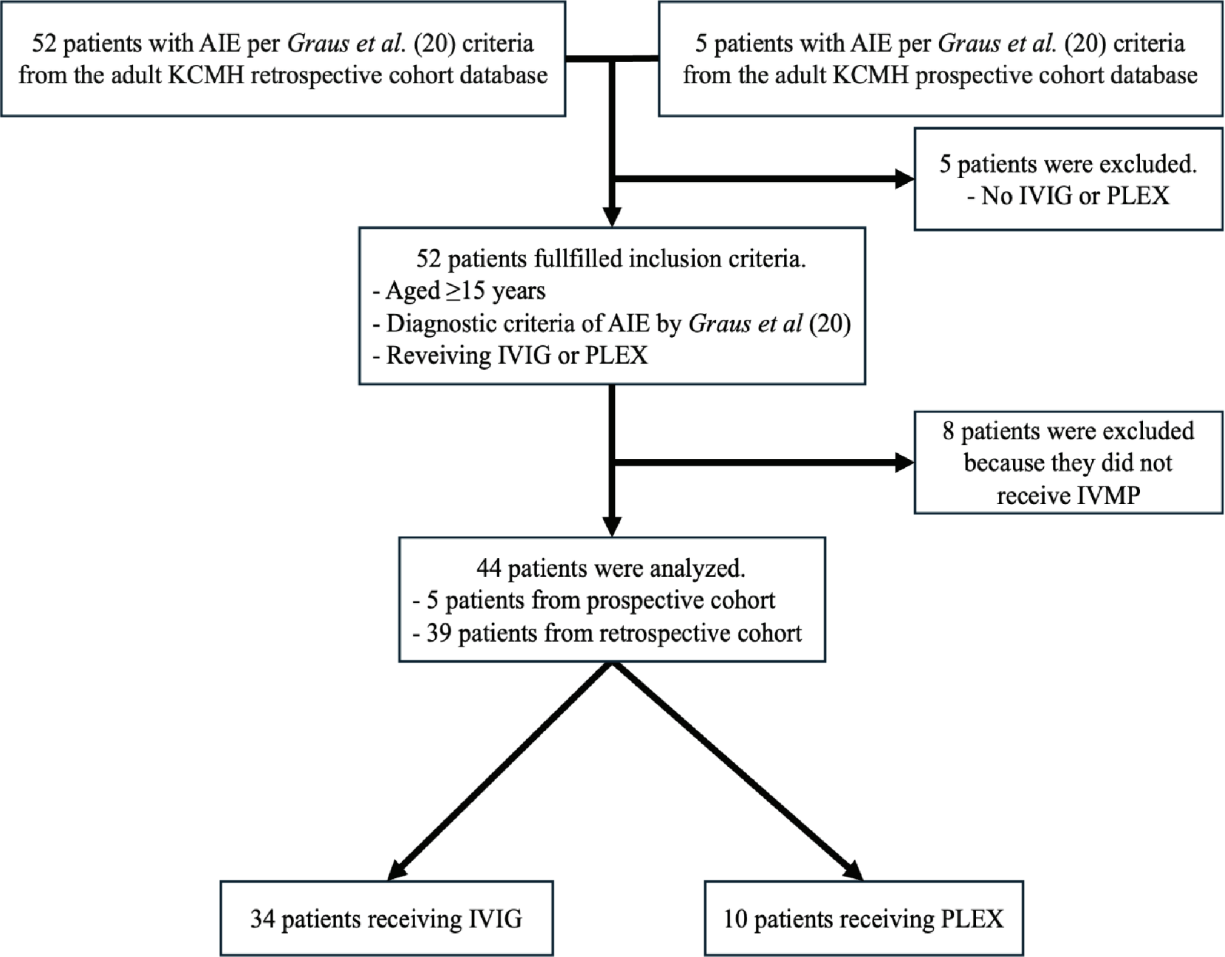
Flow diagram of inclusion and exclusion of the study. AIE, autoimmune encephalitis; IVIG, intravenous immunoglobulin; IVMP, intravenous pulse methylprednisolone; KCMH, King Chulalongkorn Memorial Hospital; PLEX, plasma exchange.

In this study, 5 patients were excluded for not receiving IVIG or PLEX as required by the inclusion criteria, and an additional 8 patients were excluded for not receiving IVMP, as shown in **Figure 1**. There were 5 patients from the prospective cohort and 39 patients from the retrospective cohort. Of all 44 patients who were hospitalized due to AIE (70.5% female; median [IQR] age and mRS at admission of 44.5 [28.0–62.5] years and 3.0 [2.0–3.0], respectively), 10 (22.7%) received PLEX and 34 (77.3%) received IVIG. There were no statistically significant differences in the proportion of females (80% vs. 67.7%, *P* = 0.70), median age (37.5 [24.0–55.0] vs. 50.5 [28.0–70.0] years, *P* = 0.14), and median mRS at admission (3.0 [3.0–3.0] vs. 3.0 [2.0–3.0], *P* = 0.65) between the two groups (**Table 1**). There were 7 (15.9%) patients who had underlying cancer: 1 (10%) in PLEX and 6 (17.7%) in the IVIG group, 3 of whom had advanced-stage cancer.

**Table 1.** Baseline characteristics of 44 patients hospitalized due to AIE at the King Chulalongkorn Memorial Hospital, The Thai Red Cross Society, between January 2015 and December 2019

	**Total (n = 44) n (%)**	**IVIG group (n = 34) n (%)**	**PLEX group (n = 10) n (%)**	***P***
Median (IQR) age, years	44.5 (28.0–62.5)	50.5 (28.0–70.0)	37.5 (24.0–55.0)	0.14
Gender				0.70
Male	13 (29.5)	11 (32.4)	2 (20.0)	
Female	31 (70.5)	23 (67.7)	8 (80.0)	
mRS at admission				0.43
Median (IQR)	3.0 (2.0–3.0)	3.0 (2.0–3.0)	3.0 (3.0–3.0)	0.65
2	14 (31.8)	12 (35.3)	2 (20.0)	
3	27 (61.4)	19 (55.9)	8 (80.0)	
4	3 (6.8)	3 (8.8)	0 (0)	
Cancer	6 (13.6)	5 (14.7)	1 (10.0)	>0.99
Diabetes mellitus	6 (13.6)	5 (14.7)	1 (10.0)	>0.99
Cirrhosis	1 (2.3)	1 (2.9)	0 (0)	0.77
Currently using immunosuppressive drugs^†^	3 (6.8)	3 (8.8)	0 (0)	>0.99
Place of admission				0.13
ICU	3 (6.8)	1 (2.9)	2 (20.0)	
Regular ward	41 (93.2)	33 (97.1)	8 (80.0)	
Type of antibodies				0.61
Intracellular	14 (31.8)	12 (35.3)	2 (20.0)	
Neuronal surface	24 (54.6)	18 (52.9)	6 (60.0)	
Ab negative	6 (13.6)	4 (11.8)	2 (20.0)	

† Immunosuppressive drugs are the prior use of steroid-sparing immunosuppressants or high-dose corticosteroids [22] including prednisolone of >40 mg/d or equivalent dose which did not include treatment with IVMP during admission. The details of the 3 patients are as follows: azathioprine, azathioprine plus prednisolone 60 mg/d, and tacrolimus plus mycophenolate mofetil.

AIE, autoimmune encephalitis; ICU, intensive care unit; IQR, interquartile range; IVIG, intravenous immunoglobulin; IVMP, intravenous pulse methylprednisolone; mRS, modified Rankin Scale; PLEX, plasma exchange.

**Figure 2.**
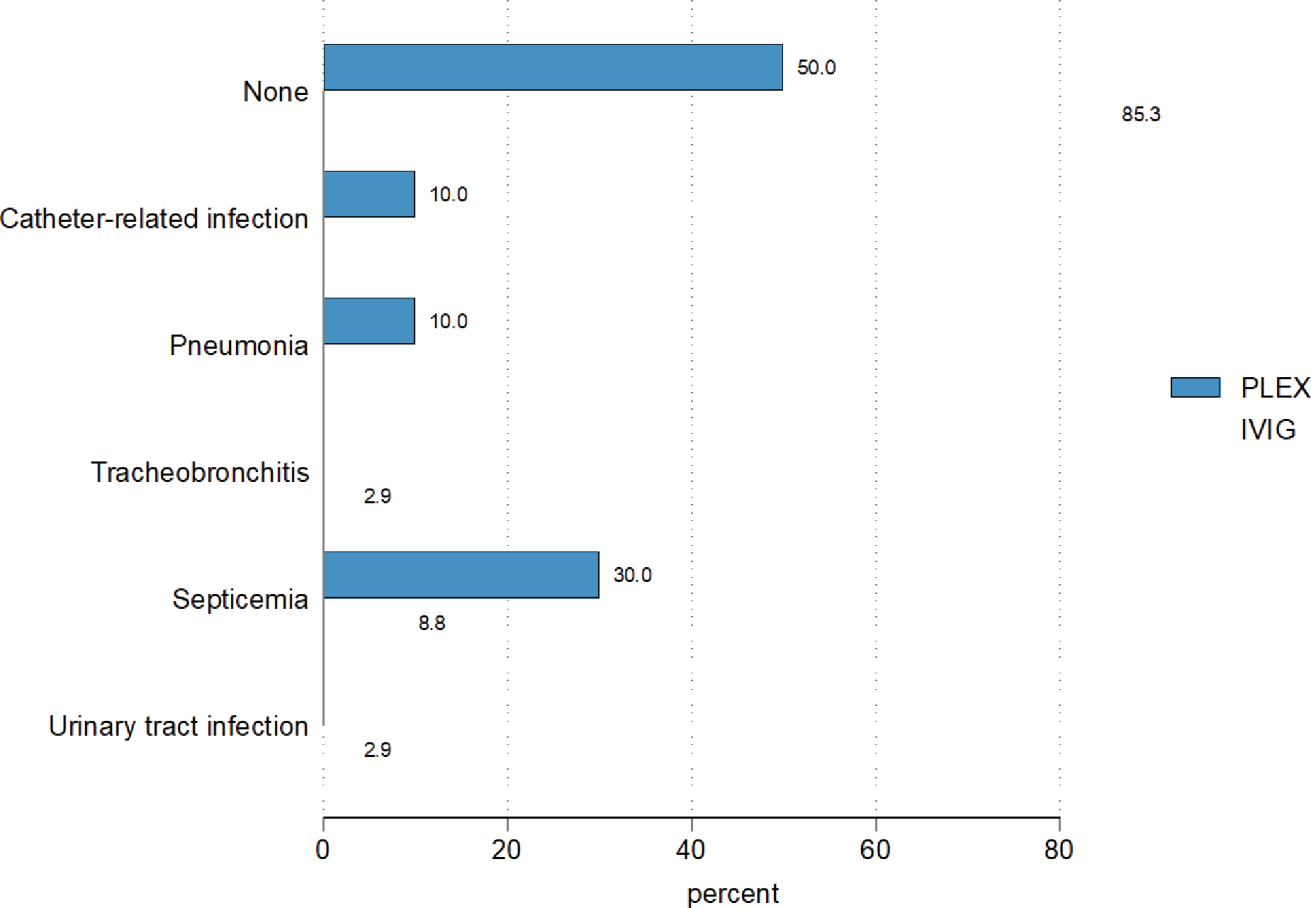
Cumulative incidence of HAIs of patients hospitalized due to AIE, categorized by those who received PLEX and IVIG. AIE, autoimmune encephalitis; HAIs, hospital-acquired infections; IVIG, intravenous immunoglobulin; PLEX, plasma exchange.

Ten participants developed HAIs after treatment (5 per group). Those who received IVIG were significantly less likely to develop HAIs compared with those who received PLEX (adjusted odds ratio: 0.17, 95% CI 0.03–0.85, *P* = 0.031) as shown in **Table 2**and **Figure 2**. The leading cause of HAIs in both groups was primary septicemia. A solitary case of catheter-related infection was observed in patients receiving PLEX, necessitating the administration of low-molecular-weight heparin and resulting in an extended hospital stay. Most infections were of mild-to-moderate severity (50%). There were three cases with severe infections (two in the IVIG group and one in the PLEX group). Infectious pathogens included *Escherichia coli* ESBL, *Klebsiella pneumoniae* CRE, *Pseudomonas Aeruginosa*, and *Staphylococcus aureus*. The most common pathogen identified in blood cultures was *K.pneumoniae* CRE in the IVIG group and *P. aeruginosa* in the PLEX group. Catheter-related infection was found in one (10%) patient in the PLEX group. In the IVIG group, one patient died due to septic shock caused by *K. pneumoniae* CRE septicemia. In the PLEX group, one patient died due to septic shock caused by *P. aeruginosa* septicemia, complicated with empyema thoracis and a right thalamic abscess.

**Table 2.** Occurrence of infectious complication during hospitalization, duration of hospitalization, degree of disability at discharge, and total direct cost of hospitalization of patients hospitalized due to AIE

	**Total (n = 44) n (%)**	**IVIG group (n = 34) n (%)**	**PLEX group (n = 10) n (%)**	***P***
HAI	10 (22.7)	5 (14.7)	5 (50.0)	0.03^*^
Duration of hospitalization (d)				0.05
≤7	11 (25.0)	11 (32.5)	0 (0)	
8–30	24 (54.5)	18 (52.9)	6 (60)	
>30	9 (20.5)	5 (14.7)	4 (40)	
Median (IQR)	16.5 (7.5–28.0)	12.0 (6.0–23.0)	25.0 (21.0–49.0)	0.01^*^
mRS at discharge				0.31
0	1 (2.3)	1 (2.9)	0 (0)	
1	20 (45.5)	17 (50.0)	3 (30.0)	
2	19 (43.2)	14 (41.2)	5 (50.0)	
3	1 (2.3)	1 (2.9)	0 (0)	
5	1 (2.3)	0 (0)	1 (10.0)	
6	2 (4.5)	1 (2.9)	1 (10.0)	
Median (IQR)	2.0 (1.0–2.0)	1.0 (1.0–2.0)	2.0 (1.0–2.0)	0.12
Median (IQR) of differences in mRS between admission and discharge	−1.0 (−2.0 to −1.0)	−1.0 (−2.0 to −1.0)	−1.0 (−2.0 to −1.0)	0.57
Median (IQR) total direct cost of hospitalization, USD^†^	8,063.9 (6,378.9–11,149.3)	8,322.9 (6,698.8–10,402.2)	7,024.8 (6,206.1–12,838.0)	0.54

† Conversion rate: 35 Thai Baht is approximately US$1.

* Statistically significant, *P* < 0.05.

AIE, autoimmune encephalitis; HAI, hospital-acquired infection; IVIG, intravenous immunoglobulin; IQR, interquartile range; mRS, modified Rankin Scale; PLEX, plasma exchange.

In addition, patients who received IVIG experienced a significantly shorter duration of hospitalization compared with those who received PLEX (12 [6–23] vs. 25 [21–49] d, *P* = 0.01), even though there were no significant differences in mRS at discharge, the change in mRS from admission to discharge, or the total direct cost of hospitalization.

## Discussion

This study demonstrated that the patients who were hospitalized due to AIE and treated with IVIG had significantly lower occurrence of HAIs and a shorter duration of hospitalization compared to those who were treated with PLEX, with comparable mRS at discharge and total direct cost of hospitalization compared with those who received IVIG. The cumulative incidence of HAIs in this study was higher compared to a previous report from KCMH that reported a 2.96% rate of catheter-related bloodstream infection (CRBSI) and a 1.89% rate of exit-site infection [23].

The median duration of hospitalization for the IVIG group was 12 d, while the PLEX group was 25 d, almost double the length of hospitalization. Longer hospital stays may lead to HAIs, which in turn can lead to longer hospitalization and vice versa [24,25,26]. In general, PLEX needs to be run 5–7 times every other day, so hospitalization takes longer (at least 10–14 d). Unlike PLEX, IVIG can be administered and completed within approximately 5 d. Nevertheless, even hospitalization after a full course of therapy was significantly different between the IVIG and PLEX groups (11 d and 16 d, respectively), as well as the rate of HAIs. This may suggest that HAIs are one of the important causes of prolonged hospital stays in the PLEX group, regardless of the duration of the treatment procedure.

Patients with intracellular antibodies had significantly longer hospital stays after receiving PLEX than IVIG. Thus, there was no difference in patients with neuronal surface antibodies. This could be due to the long stay of one patient in the IVIG group who was hospitalized for 174 d due to multiple severe infections, including a liver abscess, recurrent 5 episodes of primary septicemia from *K. pneumoniae* CRE.

Importantly, this study demonstrated comparable mRS at discharge and total direct cost of hospitalization between the two groups, despite significant differences observed in HAIs occurrence and duration of hospitalization. This aligns with previous studies that found no difference in efficacy between PLEX and IVIG [5,6,7]. However, it is crucial to note that our data collection did not account for the indirect costs, such as the loss of productivity and absenteeism of the patients and their families, as well as the opportunity cost for what healthcare providers have to provide, particularly given the significant differences in the rate of HAIs and duration of hospitalization between the two treatments.

To our knowledge, this study is among the first to compare HAIs and hospital stays in patients with AIE receiving IVIG or PLEX. However, certain limitations must be considered. First, the sample size was small, limiting our ability to conduct statistical models to adjust for potential confounders. Second, the fact that all patients were from a single site, a tertiary care center, may limit the generalizability of our findings and cause referral bias. Third, the cost variables, we were able to collect only encompassed the total direct cost of each patient’s hospitalization. Therefore, we cannot account for several indirect costs. Another limitation is the number of patients receiving IVIG is more than the number of patients receiving PLEX because the patient’s family usually prefers a noninvasive procedure to an invasive procedure. The situation can cause self-selection bias. The lack of follow-up data also limited our ability to assess long-term clinical outcomes. Regardless, we firmly believe that we were able to achieve our main objectives of providing information on the differences in multiple outcomes between the use of PLEX and IVIG for patients hospitalized with AIE, thereby filling a gap in knowledge given the challenges in diagnosing the disease, particularly in the Asia region. Future studies with large sample sizes need to be further investigated.

Even though the same outcomes were obtained, we concluded that in patients with AIE, first-line treatment with PLEX resulted in longer hospital stays and increased HAIs rates compared with IVIG. The longer the hospital stay due to PLEX procedure, the more the risk of infection may accumulate. Furthermore, longer patient stays and HAIs will certainly increase the cost of treatment. Therefore, when economic factors are taken into account, IVIG may be more cost-effective than PLEX. This was especially true in hospitals where hospitalization costs are high.
